# Alfred M‘Clintock, Esq., on the Employment of the Chloride of Zinc as an Escharotic

**Published:** 1842-06-25

**Authors:** Alfred M‘Clintock


*On the Employment of the Chloride of Zinc as an Escharotic.* By Alfred M'Clintock, Esq.—"The following cases, in which the chloride of zinc was employed, occurred in the County Louth Infirmary, under the care of Dr. Brunker, by whose permission I am enabled to offer them to the readers of the Dublin Medical Journal. The manner in which the chloride of zinc was used was similar to that employed by M. Conquoin, who first introduced this remedy into practice: one part by weight of the chloride, and two parts of flour were mixed together by adding a sufficient quantity of water to form them into a paste; this was spread over the entire surface of the diseased part, care being taken to prevent it coming into contact with the healthy structures in the neighbourhood; a piece of dry lint was then laid on, and lastly, a piece of thin bladder, moistened, was placed over all and secured with strips of adhesive plaster. The patients were confined to their ward but not to bed.

"Case I.—John Maguinness, ætat. 55, a stout, healthy countryman, was admitted 19th May, 1840, with a cancerous tumour, of a globular form, and about the size of a walnut, situated on the superior part of the pinna of the right ear; states that it began like a wart nearly four years ago, since which time it has gradually been increasing, and has become the source of much pain and annoyance. Its surface presents no peculiarity beyond what is usually observed in cancerous tumours, namely, being rugged, slightly fissured, and of a dirty brown colour, hard to the touch, and firmly attached to the subjacent parts. He got a purgative draught upon admission, and on the following day the paste was applied in the manner already described, over the entire extent of the morbid growth.

"21st. Complains of a great increase of pain, which he says deprived him of nearly all rest. No acceleration of pulse—some redness immediately about the base of the tumour.

"22d. Pain much less, somewhat more redness.

"23d, 24th. Pain continues to diminish; a small line of separation beginning to form around the attachment of the tumour.

"25th. (Fifth day since application of paste.) The slough came away to-day in a dry shrivelled state, except at the surface of the attachment, and bringing with it the entire of the disease. The ulcer left presented a healthy appearance, and was simply dressed with dry lint; the processes of granulation and cicatrization went on very favourably up to the 4th June, at which time he left the hospital; however, only a very small portion of the ulcer remains uncicatrized. The shape and figure of the ear are not, in any way, altered.

"The second case was in a man, ætat. 53, who had an ulcer rather larger than a sixpence, nearly circular, and having rounded edges, its surface smooth and glazed, of a dusky red hue, and destitute of any distinct granulations. It was not painful, and very slow in its progress, and had resisted various treatments. A thin stratum of the chloride of zinc paste was spread over it. During that night and the following day he suffered great pain. The slough separated on the sixth day, leaving a small portion of the bone exposed, the exfoliation of which protracted the healing of the part.

"In the third case the man was 57, of a healthy constitution, having a cancerous tumour on the left side of the nose, of three years' standing. He suffered lancinating pains in it. The base of the tumour was as large as a fourpenny, its surface elevated and convex, of a dirty brown colour, and rough, the attachment to the subjacent parts firm, no discolouration of the surrounding skin. He suffered pain for near three days after the application of the paste, and suffered slight constitutional disturbance, marked by rigor and nausea. The slough separated on the fifth day. The sore was healed on the eighteenth day.

"These two last cases have since been under Mr. M'Clintock's observation, and there has been no return of the disease. He has lost sight of the other case."

In commenting upon the result of the above cases, he remarks:

"1st. That in each of them the application was productive of much pain, which lasted for twenty-four or forty-eight hours, after which it began to diminish.

"2d. In only one instance, Hanlon's case, were there any symptoms that could be considered indicative of constitutional disturbance, and they were such as generally usher in an attack of erysipelas; such, however, did not supervene, as these unpleasant symptoms disappeared under the use of simple remedies.

"3d. In two of the cases the slough separated on the fifth day, and in the other on the sixth. The ulcer left, in each instance, was remarkably healthy, and cicatrized rapidly; so far confirming Dr. Ure's account of this escharotic in the 'Cyclopœdia of Practical Surgery' (Art. 'Caustics.')

"4th. The action of the chloride in both the cases of cancer was exclusively confined to the morbid structure, and destroyed it to its entire extent. In contemplating these two facts, the conclusion is forced upon our mind, that the chloride of zinc exerts a specific action on the cancerous growth."

*Dublin Jour. Med. Sci.* May, 1842.

